# Up-Regulated Expression of Pro-Apoptotic Long Noncoding RNA lincRNA-p21 with Enhanced Cell Apoptosis in Lupus Nephritis

**DOI:** 10.3390/ijms22010301

**Published:** 2020-12-30

**Authors:** Yi-Cheng Chen, Pin-Yu Kuo, Yu-Chi Chou, Hao-Earn Chong, Yu-Tung Hsieh, Mei-Lin Yang, Chao-Liang Wu, Ai-Li Shiau, Chrong-Reen Wang

**Affiliations:** 1 Department of Internal Medicine, National Cheng Kung University Medical College and Hospital, Tainan 70403, Taiwan; s58971081@gs.ncku.edu.tw (Y.-C.C.); haoen1986@gmail.com (H.-E.C.); 2 Department of Biochemistry and Molecular Biology, National Cheng Kung University Medical College, Tainan 70101, Taiwan; wumolbio@mail.ncku.edu.tw; 3 Department of Microbiology and Immunology, National Cheng Kung University Medical College, Tainan 70101, Taiwan; fishwukuo012057@gmail.com (P.-Y.K.); c216439@gmail.com (Y.-T.H.); ornas331@yahoo.com.tw (M.-L.Y.); alshiau@mail.ncku.edu.tw (A.-L.S.); 4 Biomedical Translation Research Center, Academia Sinica, Taipei 11529, Taiwan; chou0315@gate.sinica.edu.tw

**Keywords:** lupus nephritis, cell apoptosis, lincRNA-p21, CRISPR interference

## Abstract

Accelerated cell apoptosis with dysregulated long noncoding RNAs is the crucial pathogenesis in lupus nephritis (LN). Pro-apoptotic lincRNA-p21 was studied in LN patients, cell lines with lentivirus-mediated overexpression and CRISPR interference (CRISPRi)-conducted repression, and a mouse model. Clinical samples were from patients and age/sex-matched controls. Expression of lincRNA-p21 and endogenous RNA target miR-181a, were examined in mononuclear and urine cells. Guide RNA sequences targeting lincRNA-p21 were cloned into CRISPRi with dCas9/ Krüppel-associated box (KRAB) domain. LincRNA-p21-silened transfectants were investigated for apoptosis and miR-181a expression. LincRNA-p21-overexpressed cells were evaluated for apoptosis and p53-related down-stream molecules. Balb/C mice were injected with pristane to induce LN and examined for apoptosis and lincRNA-p21. Higher lincRNA-p21 levels were found in LN mononuclear and urine cells, positively correlated with activity. There were lower miR-181a levels in LN mononuclear cells, negatively correlated with activity. Doxorubicin-induced apoptotic cells had up-regulated lincRNA-p21 levels. CRISPRi with dCas9/KARA domain showed efficient repression ability on transcription initiation/elongation. CRISPRi-conducted lincRNA-p21-silenced transfectants displayed reduced apoptosis with up-regulated miR-181a levels, whereas lentivirus-mediated lincRNA-p21-overexpressed cells revealed enhanced apoptosis with up-regulated downstream PUMA/Bax expression. LN mice had glomerular apoptosis with progressive increased lincRNA-p21 levels. Our results demonstrate up-regulated lincRNA-p21 expression in LN, implicating a potential diagnostic marker and therapeutic target.

## 1. Introduction

Systemic lupus erythematosus (SLE) is an autoimmune disease characterized by a loss of immune tolerance with the formation of immune complexes (IC) containing nuclear autoantigens, resulting in inflammation at various organs and tissues, of which damage to the kidney as a consequence of lupus nephritis (LN) is the most common cause of morbidity [1,2]. The current understanding of the crucial pathogenesis involves an imbalance between production of apoptotic cells and disposal of apoptotic materials [3]. Furthermore, apoptotic cell death with inefficient clearance results in the accumulation of self-double strand DNA (dsDNA), followed by a break of tolerance to induce production of dsDNA antibody with IC deposits in glomerular and tubular basement membranes, contributing to the development of LN [3,4]. Notably, increased apoptotic T cells in lupus patients has been demonstrated to be correlated with higher disease activity [5]. A therapeutic strategy to inhibit apoptotic process, can not only reduce nuclear autoantigens production and avoid the formation of IC, but also suppress apoptotic death of phagocytes to restore their clearance of apoptotic remnants [3]. 

Long noncoding RNAs (lncRNAs) can modulate a variety of cellular processes and activities through chromatin remodeling, epigenetic modification, and gene transcription by their interaction with other intra-nuclear molecules [6]. Extensive functional potentials of lncRNAs have been elucidated in autoimmune disorders [7]. Up- or down-regulated expression of certain lncRNAs has been reported to be correlated with disease activity in LN, and these molecules participate in targeting signaling transduction such as the p53 gene pathway [8]. Lupus patients had higher p53 expression in circulating lymphocytes with a positive correlation with clinical activity, suggesting its pathogenic role in inducing apoptotic death of lymphocytes [9]. LincRNA-p21 is involved in the regulation of cell apoptosis with an action mechanism through p21, a cell-cycle gene [10]. This lncRNA is directly induced by p53 through binding to its promoter with transcriptional activation. Upon DNA damage, decreased apoptosis has been identified in lincRNA-p21-depleted primary cells, whereas lincRNA-p21-overexpressed tumor cells had increased apoptotic death [11]. Interestingly, reduced lincRNA-p21 levels in rheumatoid arthritis contribute to increased activity due to physically sequestrating NF-κB p65 mRNA by this molecule [12]. LncRNAs with microRNAs (miRNAs) response elements (MREs), can act as competing endogenous RNAs (ceRNAs) to degrade miRNAs and compete for the mRNA binding [13]. LincRNA-p21, with MREs for miR-181 members, has been demonstrated to serve as a ceRNA [10]. Although the expression of miRNAs is highly responsive to the cytokine environment [14], miRNA-181a levels were shown to be down-regulated in circulating mononuclear cells (MNCs) from SLE [15], a disease with increased expression of abundant cytokines [16]. In sum, these findings suggest a pathogenic role of lncRNA-p21 in active SLE with accelerated cell apoptosis.

Clustered regularly interspaced short palindromic repeat (CRISPR) gene editing with active Cas9 can cause an irreversible DNA cleavage, whereas CRISPR interference (CRISPRi)-based genetic perturbation with catalytically dead Cas9 (dCas9) reversibly represses target genes by inhibiting both of the initiation and elongation processes in transcription [17]. Furthermore, coupling dCas9 with Krüppel-associated box (KRAB) repressor to attract epigenetic modifiers and chromatin remodelers can fully block the transcriptional activity with a therapeutic potential in various disease states [18].

In this study, we investigated the expression of lncRNA-p21, H19, a lncRNA counteracting apoptosis through down-regulating p53 expression, and miR-181a in circulating MNCs and urine cells from LN patients, and human kidney and T-lymphocyte cell lines. Lentiviral vector (LV)-mediated overexpression of lincRNA-p21 in HK-2 cells were examined for cell apoptosis, caspase 3 and p21 levels and expression of p53-related downstream molecules. We first confirmed the efficient transcription repression ability of pAll-dCas9-KRAB.pPuro, an all-in-one CRISPRi vector with a dCas9/KRAB domain. CRISPRi-conducted lincRNA-p21-silenced HEK 293T and Jurkat transfectants were created for investigating cell apoptosis, caspase 3 and p21 levels, as well as miR-181a expression. A pristane-induced LN mouse model was studied for in situ apoptosis and levels of anti-DNA, proteinuria, and lincRNA-p21.

## 2. Results

### 2.1. Up-Regulated Expression of LincRNA-p21 in LN Patient

**Figure 1:**
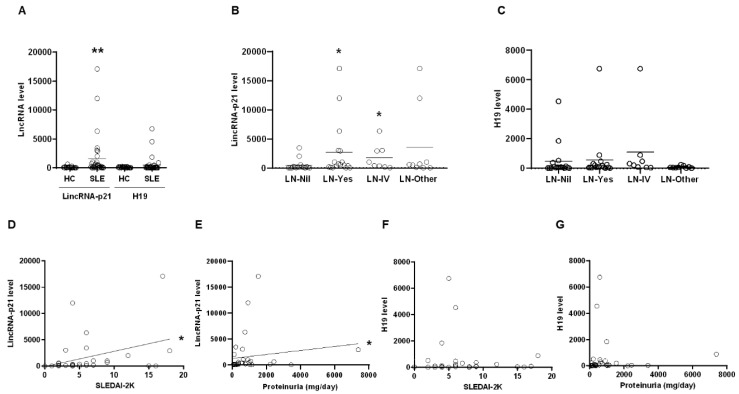
Up-regulated expression of lincRNA-p21 in PBMNCs from systemic lupus erythematosus (SLE) and lupus nephritis (LN) patients. (**A**) LincRNA-p21 and H19 levels in healthy controls (HCs) and SLE patients. (**B**) LincRNA-p21 levels in SLE patients without renal involvement and LN patients, including IV histopathology and others. (**C**) H19 levels in SLE without renal involvement and LN patients including IV histopathology and others. (**D**,**E**) A significant positive correlation between lincRNA-p21 levels and SLEDAI-2K scores or daily proteinuria amounts in SLE patients. (**F**,**G**) No correlation between H19 levels and SLEDAI-2K scores or daily proteinuria amounts in SLE patients. Horizontal lines in (**A**–**D**) are mean values from HCs and patients. *n* = 30 for HCs, *n* = 34 for SLE patients, *n* = 17 for LN patients, *n* = 17 for SLE without renal involvement, *n* = 8 for LN patients with IV histopathology, and *n* = 9 for other LN patients. * *p* < 0.05, ** *p* < 0.01.

Firstly, we examined MMCs from SLE patients and healthy controls (HCs) for the expression of lincRNA-p21 and H19. Significantly higher lincRNA-p21 rather than H19 levels were found in SLE patients in comparison with HCs (Figure 1A, *p* = 0.002). LN patients or those with class IV histopathology, had higher levels of lincRNA-p21 than those without renal involvement (Figure 1B, LN versus Nil, *p* = 0.013, LN-IV versus Nil, *p* = 0.016). There were no differences in H19 levels between SLE patients without renal involvement and those with LN, either class IV histopathology or others (Figure 1C). Moreover, there was a significant positive correlation between lincRNA-p21 levels and SLEDAI-2K scores (Figure 1D, *r* = 0.423, *p* = 0.013) or daily proteinuria amounts (Figure 1E, *r* = 0.395, *p* = 0.021). No correlation was identified between H19 levels and SLEDAI-2k scores or proteinuria amounts (Figure 1F,G).

**Figure 2:**
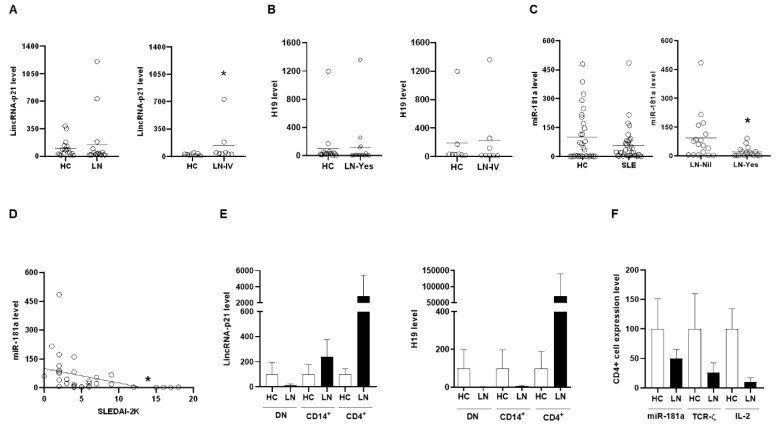
Up-regulated expression of lincRNA-p21 in urine cells and down-regulated expression of miR-181a in PBMNCs from LN patients. (**A**) LincRNA-p21 levels in urine cells from HCs and LN patients or IV histopathology. (**B**) H19 levels in urine cells from HCs and LN patients or IV histopathology. (**C**) miR-181a expression in PBMNCs from HCs and SLE patients, and from SLE patients without renal involvement and LN patients. (**D**) A significant negative correlation between miR-181a levels and SLEDAI-2K scores. (**E**) LincRNA-p21 and H19 levels in PBMNCs subpopulations including DN, CD14+ and CD4+ cells from HCs and LN patients. (**F**) MiR-181a, TCR-ζ chain, and IL-2 levels in CD4+ cells from HCs and LN patients. Horizontal lines in (**A**–**C**) are mean values from HCs and patients. Data are expressed in mean with SEM in (**E**,**F**). *n* = 30 for HCs and *n* = 34 for SLE patients (in PBMNCs), *n* = 17 for HCs and LN patients (in urine cells), *n* = 8 for HCs and IV histopathology (in urine cells), *n* = 3 for HCs and LN patients (in PBMNCs subpopulations), and *n* =3 for HCs and LN patients (in CD4+ cells). DN: double negative. * *p* < 0.05.

In Figure 2A, there were higher lincRNA-p21 levels in urine cells from LN patients than HCs (LN versus HC, 150.4 ± 78.0 versus 100.0 ± 27.1 %), and patients with class IV histopathology had significantly higher lincRNA-p21 levels than HCs (*p* = 0.028). No differences were found in H19 levels between HCs and LN patients or those with class IV histopathology (Figure 2B). Next, we analyzed the expression of miR-181a in MNCs from SLE patients. There were decreased miR-181a levels in SLE patients (Figure 2C, SLE versus HC, 56.8 ± 16.0 versus 100.0 ± 29.6 %), and LN patients had significantly lower levels than SLE without renal involvement (Figure 2C, *p* = 0.011). A significant negative correlation existed between miR-181a levels and SLEDAI-2K scores (Figure 2D, *r* = −0.383, *p* = 0.026).

MNC subpopulations from LN patients and HCs were examined for the expression of lncRNAs. CD4+T cells from LN patients had higher levels of lincRNA-p21 and H19 in comparison with HCs (Figure 2E). Interestingly, overexpressing miR-181a, an intrinsic modulator of T cell receptor (TCR) signaling, in primed T-lymphocytes can up-regulate the expression of IL-2 [19]. Lower expression of TCR-ζ chain has been identified in T cells from SLE patients with poor IL-2 production and refilling this molecule can normalize IL2 levels in vitro [20]. Further analyses of CD4+T cells from LN patients revealed lower levels of miR-181a, IL-2 and TCR-ζ chain as compared with those from HCs (Figure 2F). Collectively, these ex vivo findings from clinical samples in Figure 1 and Figure 2 demonstrated up-regulated expression of lincRNA-p21 in LN patients.

### 2.2. Transcription Repression Ability in pAll-dCas9-KRAB.pPuro with dCas9/KRAB Domain

**Figure 3:**
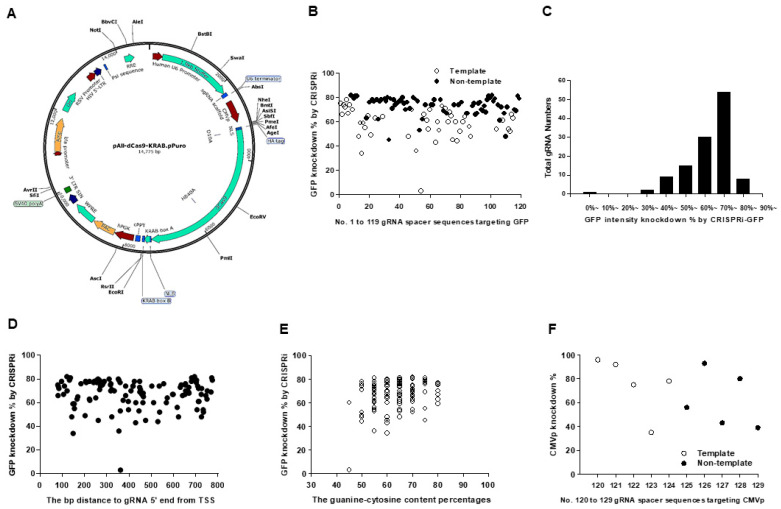
pAll-dCas9-KRAB.pPuro and GFP silencing efficacy in CRISPRi-GFP-transduced HEK-293 cells. (**A**) Map of pAll-dCas9-KRAB.pPuro with a dCas9/Krüppel-associated box (KRAB) repression domain, total 14,775 bp in length. Resistance to puromycin was conferred by the PAC gene. (**B**) GFP knockdown percentages in CRISPRi-GFP-transduced HEK-293 cells in 119 gRNA spacer sequences targeting the d2EGFP coding region. (**C**) Spike graph showing the numbers of designed gRNAs in variable GFP knockdown zones. (**D**) GFP knockdown efficacy by 119 gRNA spacer sequences with different targeting distance from TSS. (**E**) GFP knockdown efficacy and the guanine-cytosine content percentages of 119 gRNA spacer sequences. (**F**) CMVp knockdown percentages in p5w-dCas9-KRAB.pBsd- and pU6-sgRNA.Ppuro-transduced HEK-293 cells in 10 gRNA spacer sequences targeting the CMVp region. The results in (**B**–**E**) are representative of at least two independent experiments with similar findings. HA: HA tag, human influenza hemagglutinin amino acids 98–106. NLS: nuclear localization sequence. TTS: transcription start site.

pAll-dCas9-KRAB.pPuro vector is shown in Figure 3A. Green fluorescent protein (GFP) silencing effects in gRNA sequences are demonstrated in Figure 3B with more than half no less than 70% efficacy (Figure 3C). No differences were found in sequences targeting distance from TSS (Figure 3D) or guanine-cytosine contents (Figure 3E). CMV promoter silencing effects in gRNA sequences are shown in Figure 2F with more than half no less than 75% efficacy. These findings indicated that pAll-dCas9-KRAB.pPuro with dCas9/KRAB domain has efficient repression ability on transcription initiation and elongation through targeting promoter and coding regions, respectively.

### 2.3. Up-Regulated Expression of LincRNA-p21 in Apoptotic Human T-Lymphocyte and Kidney Cell Lines

**Figure 4:**
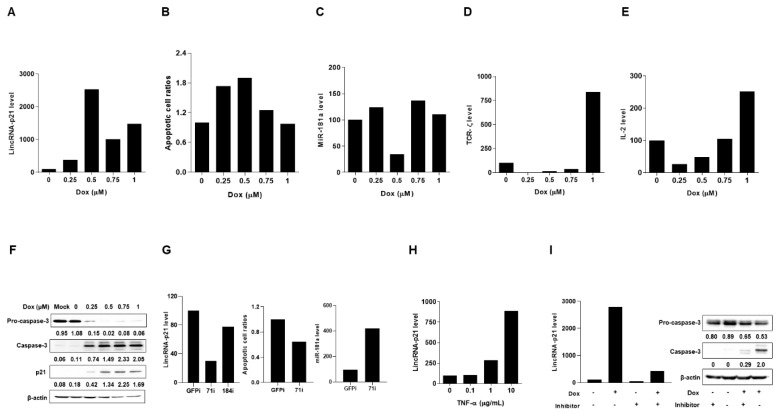
Up-regulated lincRNA-p21 expression in apoptotic T-lymphocyte cell line. (**A**–**F**) Dose-dependent up-regulated lincRNA-p21 levels, apoptotic cell ratios and caspase 3/p21 levels with reciprocal down-regulated miR-181a levels and reduced expression of TCR-ζ chain and IL-2 in Jurkat cells culture under the Dox treatment. (**G**) Two CRISPRi-lincRNA-p21 transfectants and decreased Dox-induced apoptotic cell ratios with increased miR-181a levels shown in 71i with higher silenced efficacy. (**H**) Dose-dependent increases in lincRNA-p21 levels in cell culture in the presence of TNF-α (**I**) Down-regulated expression of lincRNA-p21 in the presence of caspase 3 inhibitor in the cell culture under the Dox treatment. All of the results in Figure 4 were representative of at least two independent experiments with similar findings.

**Figure 5:**
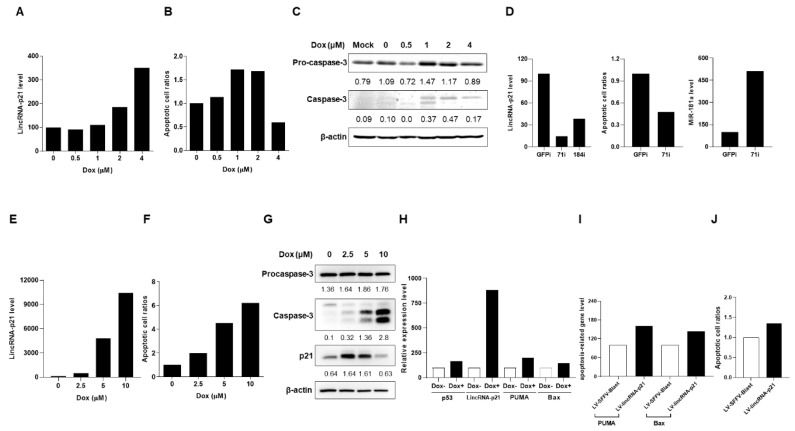
Up-regulated expression of lincRNA-p21 in apoptotic kidney cell lines. (**A**–**C**) Dose-dependent up-regulated lincRNA-p21 levels and apoptotic cell ratios and increases in caspase 3 levels in HEK 293T cells culture under the Dox treatment. (**D**) Two CRISPRi-lincRNA-p21 transfectants and decreased Dox-induced apoptotic cell ratios with increased miR-181a levels shown in 71i with higher silenced efficacy. (**E**–**G**) Dose-dependent up-regulated lincRNA-p21 levels and apoptotic cell ratios and increases in caspase 3/p21 levels in HK-2 cells under the Dox treatment. (**H**) Up-regulated expression of p53 down-stream molecules PUMA and Bax in HK-2 cells culture under the Dox 5 μM treatment. (**I**) Enhanced expression of p53 down-stream molecules PUMA and Bax and (**J**) enhanced Dox-induced apoptotic cell ratios in lincRNA-p21-overexpressed HK-2 cells. All of the results in Figure 5 were representative of at least two independent experiments with similar findings.

Under Dox-induced DNA damage to trigger p53-dependent cell apoptosis [11], we investigated lincRNA-p21 expression in a T-lymphocyte and two kidney cell lines. All of the results in Figure 4 and Figure 5 were representative of at least 2 independent experiments with similar findings.

By culturing Jurkat cells in the presence of Dox, there were dose-dependent up-regulated lincRNA-p21 levels and apoptotic cell ratios, and reciprocal down-regulation of miR-181a expression with reduced TCR-ζ chain and IL-2 levels as well as an increase in expression of caspase 3 and p21 (Figure 4A–F). Moreover, in two CRISPRi-lincRNA-p21 transfectants, the one (71i) with a higher silenced efficacy (70% knockdown in comparison with CRISPRi-GFP transfectants) had decreased Dox-induced apoptotic cell ratios and increased miR-181a levels (Figure 4G). 

Notably, TNF-α has been identified to possess the strongest correlation with SLE activity among different tested plasma cytokines [21] and regulate the expression of abundant lncRNAs [22]. LncRNA-p21 levels were up-regulated in a dose-dependent manner by adding TNF-α into Jurkat cells culture (Figure 4H). In the presence of Dox, despite a simultaneous up-regulated expression of lincRNA-p21 and caspase 3, the addition of a caspase 3 inhibitor (Z-DEVD-FMK) could reduce lincRNA-p21 levels (Figure 4I), suggesting that caspase 3 activation can provide a feedback to enhance lincRNA-p21 expression in the apoptotic cell process. 

Furthermore, we induced apoptosis in HEK 293T kidney cells with Dox treatment. There were dose-dependent up-regulated lincRNA-p21 levels, apoptotic cell ratios and caspase 3 expression (Figure 5A–C). CRISPRi-lincRNA-p21 transduced HEK 293T transfectants (71i) with a better silencing effect (86% knockdown as compared with control cells) demonstrated reduced Dox-induced apoptotic cell ratios and enhanced miR-181a expression levels (Figure 5D). 

Another renal tubular HK-2 cell line was treated with Dox to induce apoptosis, resulting in dose-dependent increases in lincRNA-p21 levels, apoptotic cell ratios, and expression of caspase 3 and p21 (Figure 5E–G). Since lincRNA-p21 can provide a feedback to enhance the p53 transcriptional activity [10], we examined the expression of p53 downstream molecules in Dox-treated HK-2 cells. There was up-regulated expression of p53, lincRNA-p21, PUMA, and Bax (Figure 5H). Moreover, lincRNA-p21-overexpressed HK-2 cells had increased expression of p53 downstream molecules PUMA and Bax (Figure 5I) with enhanced Dox-induced apoptotic cell ratios (Figure 5J). Instead of using transiently transfected cells, further studies can apply blasticidin selection process to create stable lincRNA-p21 tranfectants and examine whether there are higher expression levels of these downstream molecules. 

Altogether, these in vitro results indicated that up-regulated expression of lincRNA-p21 could enhance apoptosis in human T-lymphocyte and kidney cell lines.

### 2.4. Up-Regulated Expression of LincRNA-p21 in a LN Mouse Model

**Figure 6:**
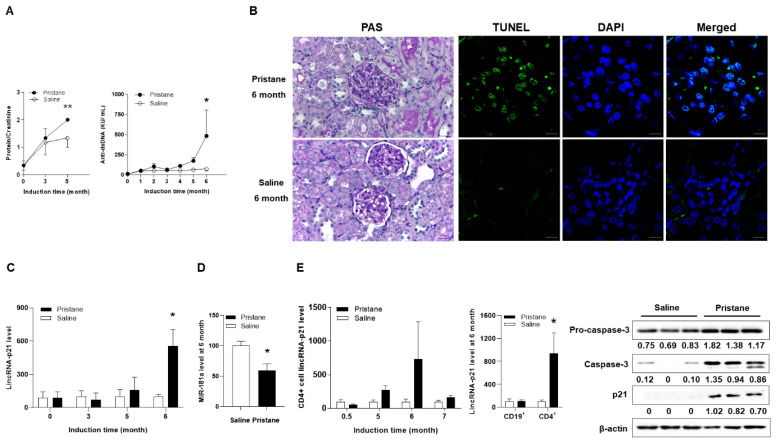
Up-regulated expression of lincRNA-p21 with in situ apoptosis in pristane-induced female Balb/C mice with LN. (**A**) Kinetic measurement of proteinuria amounts and anti-dsDNA titers after pristane induction. Values are the mean ± SEM with 3 mice per group. (**B**) PAS staining of GN with glomerular hyper-cellularity/mesangial expansion (×400) and TUNNEL staining with in situ apoptotic glomerular parenchymal cells (×200) at 6 months after induction. Saline-injected sections as the control (lower panel). Bars shown on photomicrographs at ×200 and ×400 magnification correspond to 20 μm and 10 μm, respectively. Representative microphotographs from 3 mice per group. (**C**) Kinetic measurement of lincRNA-p21 levels from kidney cells after induction. Values are the mean + SEM with 5 mice per group. (**D**) Mir-181a levels from kidney cells at 6 months after induction. Values are the mean ± SEM of 5 mice per group. (**E**) Kinetic measurement of lincRNA-p21 levels from CD4+ cells (left panel). LincRNA-p21 levels from CD19+ and CD4+ cells at 6 months after induction (middle panel), and expression of caspase 3 and p21 levels (right panel). Values are the mean ± SEM of 3 mice per group for measurement of lincRNA-p21 levels, and 3 mice per group for caspase 3/p21 immunoblot assay. All of the results in Figure 6 were representative of two independent experiments with similar findings.

Pristane-injected Balb/c mice were periodically examined for dsDNA antibody and urinary protein. Significantly elevated proteinuria amounts and higher anti-double strand DNA (dsDNA) titers were noted at 5 and 6 months after induction, respectively (Figure 6A). Their kidneys were removed upon sacrifice for histopathological and in situ apoptosis analyses. In Figure 6B, kidneys from LN mice had GN with glomerular hyper-cellularity/mesangial expansion and the presence of in situ apoptotic cells at 6 months after pristane injection, an induced model with the presence of Fas-independent transferase dUTP nick end labeling (TUNEL)-positive tissue cells [23]. Significantly up-regulated lincRNA-p21 and down-regulated miR-181a levels were shown in kidney cells from LN mice at 6 months after induction (Figure 6C,D). Furthermore, there were increasingly up-regulated lincRNA-p21 levels in CD4^+^ T cells from LN mice after pristane induction with significant higher levels at 6 months, as well as increased expression levels of caspase 3 and p21 (Figure 6E). Taken together, these ex vivo data from LN mice indicated progressively up-regulated lincRNA-p21 expression in kidney and CD4^+^ T cells.

## 3. Discussion

LN has multiple pathogenic pathways including aberrant apoptosis, autoantibody production and IC deposition with complement activation [1,2]. Apoptotic cell death with roles in tissue damage and immune dysregulation, is involved in the generation of autoantigens and the externalization of modified nuclear antigens [3]. In the development of LN, there are accelerated cell apoptosis in circulating lymphocytes [24], kidney cells (renal tubular and glomerular parenchymal cells) [25], and phagocytes for clearance of apoptotic cells [26]. LncRNAs are emerging as key players in controlling the cellular apoptotic process [6], and these molecules participate in the LN pathogenesis with aberrant expression levels [8]. In this study, up-regulated expression of pro-apoptotic lincRNA-p21, rather than anti-apoptotic H19, was identified in MNCs, especially in CD4+ T cells, from LN patients as well as a human T-lymphocyte line receiving Dox treatment to induce apoptosis. Moreover, higher lincRNA-p21 levels were detected in urine cells from LN patients and human kidney cell lines under the DNA damage response. Notably, transcriptome-wide studies have demonstrated that the expression of different lncRNAs is specific for cell types to exert their distinct regulatory functions [22]. Pro-inflammatory cytokines, TNF-α in particular, have been demonstrated to regulate the expression of lncRNAs, some of which have up-regulated levels in a NF-κB dependent manner [27], as demonstrated in this study with dose-dependent increases in lincRNA-p21 expression levels in Jurkat cells upon in vitro TNF-α stimulation (Figure 4H). Indeed, in SLE patients, elevated cytokines levels can influence the expression of lncRNAs at different tissues and organs, leading to heterogeneous clinical involvement [16,21,22]. Furthermore, we observed increases in apoptotic cell ratios and expression levels of caspase 3 and p21 in Dox-treated T-lymphocyte and kidney cell lines. Up-regulated expression of lincRNA-p21 contributes to apoptotic cell death in circulating lymphocytes and renal cells, followed by production of autoantibodies, resulting in in situ IC accumulation and the formation of GN in SLE. Further experiments can use T lymphocytes and renal tubular cells from LN patients to improve the clinical relevance of this study. 

Owing to complex disease presentations and inherent limitations in clinical research, there are difficulties in performing direct studies in lupus patients. SLE mouse models have been developed to dissect pathogenic mechanisms and identify therapeutic targets [28]. In addition to spontaneous lupus models like NZB/W F1 and MRL/lpr mice, induced mouse models, particularly the pristane-induced mice with renal IC deposition causing GN, are useful tools to investigate the molecular pathogenesis with dysregulated signaling pathways and to screen therapeutic modalities in LN [28,29]. In this study with Balb/c female mice, LN developed with increased anti-dsDNA levels, elevated proteinuria amounts, and the formation of GN after pristane induction. The pristane-induced model is driven by a strong type I IFN response [29], a well-known inducer of lncRNA expression in immune responses, which is much weaker in spontaneous mouse models like NZB/W F1 and MRL/lpr mice. Therefore, such an induced model is suitable to analyze the role of lincRNA-p21 in LN-related immune processes. Moreover, there were in situ apoptosis with up-regulated expression of lincRNA-p21 in CD4+ lymphocytes and kidney cells as well as elevated caspase 3 and p21 levels. By using the lupus mouse model through a proof-of-concept approach, we demonstrated a progressive increase in renal expression of lincRNA-p21 during the development of LN. In vitro experiments by using CRISPRi-lincRNA-p21 transfected cell lines revealed lower apoptotic cell ratios in the presence of DNA damage response, implicating a therapeutic strategy to treat LN by knocking down the expression of lincRNA-p21 to reduce cell apoptosis. Nevertheless, further efforts are needed to elucidate the potential of lincRNA-p21 as a therapeutic candidate by silencing its renal expression to examine whether there is improvement of GN in the LN mouse model. 

RNA interference can attack mature cytosolic RNA for degradation, but not effective in targeting nuclear lncRNA [30]. Despite a complete gene knockout, CRISPR/Cas9 editing can introduce the DNA cleavage with a risk of error-prone repair, whereas CRISPR/dCas9-based reversible gene repression allows specific transcriptional and epigenomic modulation at targeted loci with a less off-target effect, not only enabling the time-resolved investigation in gene functions but also predicting the outcome of pharmacological inhibition on gene products [17,30]. In this study with CRISPRi containing dCas9/KRAB repression domain for in vitro transfection, we demonstrate that silencing lincRNA-p21 inactivates p21 and caspase 3 to reduce the apoptotic cell death in human cell lines. Interestingly, dCas9 coupling a bipartite repressor KRAB-MeCP2 has recently been demonstrated to hold a higher transcription repression ability than KRAB alone [31], raising a possibility to create an all-in-one CRISPRi vector with this bipartite domain for more efficiently silencing the expression of lincRNA-p21 to enhance the inhibition efficacy on cell apoptosis. 

Transcript RNAs with specific MREs can communicate with others via the miRNA messenger and may serve as ceRNAs to de-repress the activity of other RNAs with similar MREs by competing for the same miRNAs [32]. LncRNAs harboring the MREs can serve as ceRNAs with the function to sponge or sequestrate miRNAs, and growing evidence has demonstrated that, an interaction between lncRNAs and miRNAs can regulate miscellaneous cellular processes to affect human disease states [13,32]. MiR-181a levels in PBMNCs have been shown to be down-regulated in SLE patients with higher disease activity [15], and also demonstrated in LN patients with a negative SLEDAI-2K correlation from this study. Furthermore, in cell lines under the DNA damage response, there were increases in lincRNA-p21 levels with a reciprocal decrease in miR-181a expression, and CRISPRi-mediated repression of lincRNA-p21 could restore the down-regulated expression of miR-181a. In addition, decreased mir-181a levels were observed in kidney cells form LN mice in this study. By transducing primed T cells with retroviral vector carrying miR-181a, IL-2 production can be up-regulated through targeting multiple negative regulators to augment T cell activation [19]. Impaired IL-2 production has been shown in T cells from SLE patients, and IL-2-treated LN mice have decreased autoantibody levels and reduced GN severity [33]. Notably, low-dose IL-2 treatment has recently been demonstrated to have the therapeutic efficacy in LN patients [34]. Accordingly, these findings suggest that the disease progression in LN can be reduced by inhibiting the expression of lincRNA-p21 to raise miR-181a levels for IL-2 restoration.

## 4. Materials and Methods 

### 4.1. LN Patients and Age/Sex-Matched Control Subjects 

Thirty-four patients fulfilling the American College of Rheumatology revised Criteria for SLE [35], 30 females and 4 males aged from 28 to 65 years (44.4 ± 1.6), and age/sex-matched healthy controls (HCs) were enrolled into this study. Their venous blood samples were collected for further examination. Medical records were reviewed for demographic, clinical and laboratory data, and the disease activity at the time of sample collection were assessed by SLEDAI-2K. Seventeen patients in this study had LN, 15 females and 2 males aged from 28 to 60 years (44.0 ± 2.3), including 8 with class IV, 5 with class III, 3 with class V, and one with class II histopathological findings. All SLE patients were under corticosteroids therapy. In class IV patients, 4 received cyclophosphamide, 2 used azathioprine, and 2 under mycophenolate mofetil treatment. In class III patients, 3 received azathioprine and 2 under mycophenolate mofetil therapy. Three class V patients received azathioprine therapy, and immunosuppressants were not prescribed in one class II case. In 17 SLE patients without renal involvement, 15 received azathioprine therapy, and 2 not under immunosuppressants treatment. Notably, LN patients had significantly higher SLEDAI-2K scores than SLE without renal involvement (8.4 ± 1.2 versus 3.5 ± 0.7, *p* < 0.001). Both renal involvement and kidney sparing groups has no differences in their age/sex distribution. Fresh urine specimens were collected from all LN patients and age/sex-matched HCs. This study was approved by the Institutional Review Board of National Cheng Kung University Hospital (approval number A-ER-108-455) with the informed consent from each participant.

### 4.2. Pristane-Induced LN Mouse Model

Eight-week old female BALB/c mice were purchased from the Laboratory Animal Center of our medical college, and housed under specific pathogen-free conditions. Animal experiments were approved by the Institutional Animal Care and Use Committee of our university. Mice were intraperitoneally (i.p.) singly injected with 0.5 mL pristane (Sigma-Aldrich, St. Louis, MO, USA) to induce LN, whereas the control group was i.p. singly injected with 0.5 mL of phosphate-buffered saline (PBS) [29,36]. Except 5 mice per pristane-injected or control group for measuring renal lincRNA-p21 and mir-181a levels, there were 3 mice per group in all animal experiments. Blood samples were collected monthly for examining anti-dsDNA levels until 6 months after induction, while urine specimens were harvested for measuring proteinuria concentrations at 0, 3, and 5 months. Mice were sacrificed at 0, 3, 5, and 6 months for removing the kidneys to measure lincRNA-p21/mir-181a levels and analyze histopatological findings, and at 0.5, 3, 5, 6, and 7 months for obtaining the spleens to measure lincRNA-p21 levels and perform immunoblotting assay.

### 4.3. Purification of Human and Mouse Cells 

Human MNCs were isolated from blood samples by Ficoll-Paque PLUS (GE Healthcare, Chicago, IL, USA) and incubated with CD14 microbeads. CD14+ cells were eluted from the positive selection column of Magnetic Cell Sorter (Miltenyi Biotec, Germany). CD14- cells were incubated with CD4 microbeads, and CD4+ cells were eluted from the column. Mouse spleens were homogenized by using syringe plunger and mesh strainer. Mouse MNCs were further incubated with PE-Cy5 anti-CD4 (BD Pharmingen, San Diego, CA, USA) or FITC anti-CD19 (BD Pharmingen), and sorted by Moflo XDP Cell Sorter (Beckman Coulter, Mountain View, CA) to obtain CD4+ or CD19+ cells. Purity of cell subpopulation was confirmed to be up to 95 % by flow cytometric analyses. Human urine cells were isolated from urine specimens by centrifugation and washing procedures to obtain cell pellets [37]. After removing capsules, mouse kidneys were minced into tiny pieces to obtain cortex tissues, followed by incubation with digestion buffer with collagenase (Sigma-Aldrich), and centrifuged to collect cell pellets [38]. 

### 4.4. Quantitative Real Time Polymerase Chain Reaction (qRT-PCR) 

Total RNAs from human or mouse cells were extracted by TRIzol reagent (Invitrogen, Carlsbad, CA, USA), and complementary DNAs were obtained by using reverse transcriptase (Applied Biosystems, Foster City, CA, USA). qRT-PCR was performed to quantify the target RNAs levels by using the SYBR qPCR Mix Kit (TOOLS) [39]. The condition of PCR was: 95 °C for 5 min, 95 °C for 15 s, primer-melting temperature (Tm) for 1 min with 40 cycles, and elongation at 72 °C for 20 s. Primer sequences were as follows. 

Human lincRNA-p21 (Tm 59 °C)

Forward 5′-GTGCAGAGCGTTTTGTTTGTCCAT-3′/reverse 5′-CCACAGCCTCTGGGAAG AAAATG-3′.

Human H19 (Tm 57 °C)

Forward 5′-GAAATGCTACCCAGCTCAAGC-3′/reverse 5′-CTGCTGTTCCGATGGTGTCTTTGA-3′.

Human Bax (Tm 58 °C)

Forward 5′-ATGCGTCCACCAAGAAGCTGAG-3′/reverse 5′-CCCCAGTTGAAGTTGCCATCAG-3′.

Human PUMA (Tm 58 °C)

Forward 5′-ACGACCTCAACGCACAGTACGA-3′/ reverse 5′-CCTAATTGGGCTCCATCTCGGG-3′.

Human P53 (Tm 52 °C)

Forward 5′-CCCTTCCCAGAAAACCTACC-3′/reverse 5′-CTCCGTCATGTGCTGTGACT-3′.

Human IL-2 (Tm 56 °C)

Forward 5′-CATGCCCAAGAAGGCCACAG-3′/reverse 5′-T TGCTGATTAAGTCCCTGGGTC-3′. 

Human TCR-ζ chain (Tm 55 °C)

Forward 5′-CAGCCAGGGGATTTCCACCACTC-3′ /reverse 5’-CCCTAGTACATTGACGGGTTTTTC-3′.

Human GADPH (Tm 54 °C)

Forward 5′-ACTTCAACAGCACACCCACT-3′/reverse 5′-GCCAAATTCGTTGTCATACCAG-3′.

Mouse lincRNA-p21 (Tm 57 °C)

Forward 5′-CCGACAGGAGTCTCATGCTCAG-3′/ revers 5′-CTGACCCAGACCAGTCTGGGC-3′. 

Mouse GADPH (Tm 56 °C)

Forward 5′-GTTGTCTCCTGCGACTTCAACA-3′/ reverse 5′-TTGCTGTAGCCGTATTCATTGTC-3′. 

The relative abundance of a measured gene expression was normalized by GAPDH gene from each sample. The average levels of human HCs or PBS-injected control mice, and expression levels of cell lines without stimulation, CRISPRi-GFP-silenced transfectants, and LV-SFFV-Blast-overexpressed cells were determined as 100%.

For analyzing the expression levels of human and mouse miR-181a, total RNAs were reverse transcription (RT) by using the reverse transcriptase kit (Applied Biosystems) with 10 ng purified RNA, dNTP, MultliScribe reverse transcriptase, RT buffer, RNase inhibitor, random primers, and gene-specific stem-loop RT primer with a TaqMan MicroRNA Reverse Transcription Kit (Applied Biosystems) in Smart Cycler (Cepheid, Sunnyvale, CA, USA) [39]. The reagents were incubated with 16 °C for 30 min, 42 °C for 30 min and 85 °C for 5 min. The condition of PCR was: 95 °C for 10 min, 95 °C for 15 sec and 60 °C for 1 min with 40 cycles. Quantitative expression levels of miR-181a were analyzed with RNU6B small RNA (Applied Biosystems) as an endogenous control. The average levels of human HCs or PBS-injected control mice and expression levels of cell lines without stimulation and CRISPRi-GFP-silenced transfectants were determined as 100%.

### 4.5. pAll-dCas9-KRAB.pPuro/green Fluorescence Protein (GFP) Reporter Construction

pAll-dCas9-KRAB.pPuro was constructed from pLAS2w.Ppuro (Academia Sinica, Taiwan) by inserting dCas9-KRAB fused fragment and guide RNA (gRNA)-expression cassette. dCas9 was generated by PCR amplifying the HA-NLS-Cas9-D10A nickase-NLS region from pX335 (Addgene, Watertown, MA, USA) with introduced H840A mutation. KRAB repressor was from HEK-293 cells cDNA. dCas9-KRAB fused fragment by overlapping PCR and U6 promoter-driven gRNA-expression cassette by PCR amplification, were cloned into AgeI/EcoRI and ClaI sites of pLAS2w.Ppuro, respectively. d2EGFP was cloned into NheI/PmeI sites of pLAS2w.Pbsd to create GFP reporter containing CMV promoter. 

### 4.6. Transcription Repression Assessment

To examine transcription repression on GFP coding region, 119 gRNA spacer sequences were designed with 47 template (T) and 72 non-template (NT) strands (Table 1). A 1.9 kb stuffer was removed by BsmBI to acquire CRISPRi-GFP after an overnight ligation reaction. HEK-293 cells were transfected by CRISPRi-GFP and GFP reporter mixed with TransIT-LT1 (Mirus Bio, Madison, WI, USA). GFP intensities were analyzed by ArrayScan (Thermo Scientific, Waltham, MA, USA) 24 hr after transfection with the mean of total intensity in CRISPRi mock control as 100%. To evaluate transcription repression on CMV promoter, 10 gRNA spacer sequences were designed, 5 T and 5 NT strands (Table 1). A 1.9 kb stuffer in pU6-sgRNA.Ppuro plasmid (Academia Sinica, Taiwan) was removed by BsmBI for gRNA cloning. HEK-293 cells were transfected by p5w-dCas9-KRAB.pBsd containing EF1α promoter, pU6-sgRNA.Ppuro and GFP reporter mixed with TransIT-LT1.

### 4.7. Construction of LV-Based CRISPRi Targeting and Overexpression of LincRNA-p21

CRISPRi guide RNA spacer sequences targeting human lincRNA-p21 were designed as 5′-GCAAGGCCGCATGATGATGC-3′, 71 bp from transcription start site (TTS) to 5′ end of gRNAs in template (T) strand (71i) and 5′-GCTTGCTTTGCATGATTGTT-3′, 184 bp from TTS in non-template (NT) strand (184i) [30]. A 1.9 kb stuffer was removed from pALL-dCas9-KRAB.pPuro for cloning of guide RNA spacer sequences targeting lincRNA-p21. LincRNA-p21 was generated from HEK 293T cells cDNA by PCR amplification and further cloned into LV-SFFV-Blast. To obtain CRISPRi-lincRNAp21 and LV-lincRNA-p21, the created guide RNA and lincRNA-p21 expressing vectors were transfected into sub-confluent HEK 293T cells, along with the packaging psPAX2 and envelope pMD2.G plasmids by using calcium phosphate precipitation to acquire recombinant LV [39,40]. After transfection for 48 to 72 h, cell supernatants were harvested and stored at −800 °C until use. CRISPRi-GFP and LV-SFFV-Blast vectors were used as the control vector in this study.

### 4.8. Production of Stable Transfectants

Jurkat T-lymphocyte and HEK 293T kidney cells (American Type Culture Collection, Manassas, VA, USA) with 5 × 105 cells/mL in 6-well plate, were transfected with LV-CRISPRi-lincRNA-p21 or LV-CRISPRi-GFP for 48 h in the presence of polybrene (8 µg/mL, Sigma-Aldrich), and were incubated with 5 µg/mL and 0.5 µg/mL of puromycin, respectively [40]. The puromycin selection process was up to one month in order to select successfully transduced stable transfectants confirmed by qRT-PCR analyses.

### 4.9. Doxorubicin (Dox)-Induced Cell Apoptosis

HEK 293T, HK-2, Jurkat cells, or transfectants were seeded with 1 × 106 cells/mL in 6-well plate in the presence of Dox (TTY Biopharm, Taiwan) for 24 h under 37 °C, 5 % CO_2_ incubation [11]. These cells were further stained with Annexin V and 7-AAD (BD Pharmingen) to detect apoptotic and dead cells, respectively, by flow cytometric analyses. Apoptotic cells were defined as Annexin V+ and 7-AAD- in this study. 

### 4.10. Immunoblotting Assessment

Cell lysates from human cell lines, transfectants, or mouse cells were separated by electrophoresis on 10–15 % SDS-PAGE, transferred on PVDF membranes (Merck Millipore, Burlington, MA, USA), blocked in 5 % of non-fat dry milk and incubated with primary antibodies anti-caspase 3 (Cell signaling, Danvers, MA, USA), anti-procaspase 3 (Cell signaling), anti-p21 (Santa Cruz, Santa Cruz, CA, USA), or anti-actin antibodies (Sigma-Aldrich) at 4 °C for 16–18 h [39,40]. After washing, the membranes were incubated with secondary antibodies (Jackson Immunoresearch, West Grove, PA, USA) at room temperature for 2 h. Signal expression of protein-antibody complexes was detected by ECL system (Amersham Pharmacia Biotech, Buckinghamshire, UK) and visualized with Biospectrum imaging system (UVP, Upland, CA, USA). The relative protein expressions were measured by Image J (NIH).

### 4.11. Enzyme-Linked Immunosorbent Assay (ELISA)

After pristane induction, serum samples from BALB/c mice were periodically examined for the presence of anti-dsDNA levels with an ELISA kit (Alpha Diagnosis, San Antonio, TX, USA).

### 4.12. Proteinuria Detection 

Urine samples from BALB/c mice were collected at different time periods, and proteinuria was detected by urine testing strips (Arkray, Edina, MN, USA). The results were determined by the semi-automated urine chemistry analyzer (Arkray RT-4010), and urine protein concentration (UPC) quantification data was transferred into 5 ranking including 0, 0.5, 1, 2, and 3.

### 4.13. Histopathological and Immunofluorescence Analyses 

Paraffin-embedded sections were de-paraffinized in xylene, dehydrated in ethanol and rehydrated in distilled water. To determine glomerulonephritis (GN), mouse kidney sections were analyzed by Periodic acid-Schiff (PAS) staining. For terminal deoxynucleotidyl transferase dUTP nick end labeling (TUNEL) assay to detect in situ apoptosis, antigens in the kidney sections were reactivated by proteinase K for 10 min, re-fixed by 4 % formaldehyde for 25 min, incubated with equilibrate buffer for 7 min, and finally labelled by the TUNEL detection cocktail (Promega, Madison, WI, USA) [40].

### 4.14. Statistical Analyses

Data are expressed as the mean ± standard error of the mean (SEM). The expression levels of mRNA between patients and HCs or different groups of patients were analyzed by Mann-Whitney *U*-test. Correlation analysis was analyzed by Spearman correlation coefficient test with linear regression analysis. The significant differences in other in vitro analyses were determined by Student’s *t*-test. The differences at different time points in in vivo study were analyzed by repeated-measures analysis of variance. *p* Values less than 0.05 is considered to be significant in this study with the symbols presenting as * for *p* < 0.05, ** for *p* < 0.01, and *** for *p* < 0.001.

## 5. Conclusions

A better understanding of the LN pathogenesis can help to develop disease biomarkers in diagnosis and prognosis, and to identify novel therapeutics other than conventional immunosuppressive agents with significant failures and adverse effects. In this study, by using clinical samples, human cell lines, and a mouse model, we demonstrate up-regulated expression of lincRNA-p21 in LN, implicating this pro-apoptotic lncRNA as a potential diagnostic biomarker and therapeutic target. 

## Figures and Tables

**Table 1:** gRNA space sequences targeting d2EGFP coding region (No. 1 to 119) and CMV promoter (No. 120 to 129).

No.	gRNA Spacer Sequence 5′–3′	Strand	No.	gRNA Spacer Sequence 5′–3′	Strand
1	AAGGGCGAGGAGCTGTTCAC	T	66	GCTGAAGGGCATCGACTTCA	T
2	AGGGCGAGGAGCTGTTCACC	T	67	CAGCTCGATGCGGTTCACCA	NT
3	GGGCGAGGAGCTGTTCACCG	T	68	GAAGGGCATCGACTTCAAGG	T
4	CGAGGAGCTGTTCACCGGGG	T	69	TCAGCTCGATGCGGTTCACC	NT
5	CACCGGGGTGGTGCCCATCC	T	70	GGCATCGACTTCAAGGAGGA	T
6	GGTGCCCATCCTGGTCGAGC	T	71	CGATGCCCTTCAGCTCGATG	NT
7	CCCATCCTGGTCGAGCTGGA	T	72	CAAGGAGGACGGCAACATCC	T
8	GACCAGGATGGGCACCACCC	NT	73	AAGGAGGACGGCAACATCCT	T
9	GAGCTGGACGGCGACGTAAA	T	74	AGGAGGACGGCAACATCCTG	T
10	CCGTCCAGCTCGACCAGGAT	NT	75	CAACATCCTGGGGCACAAGC	T
11	GCCGTCCAGCTCGACCAGGA	NT	76	TGTACTCCAGCTTGTGCCCC	NT
12	CGTCGCCGTCCAGCTCGACC	NT	77	CAGCCACAACGTCTATATCA	T
13	GGCCACAAGTTCAGCGTGTC	T	78	ATGGCCGACAAGCAGAAGAA	T
14	CAAGTTCAGCGTGTCCGGCG	T	79	CGGCCATGATATAGACGTTG	NT
15	AAGTTCAGCGTGTCCGGCGA	T	80	CAAGCAGAAGAACGGCATCA	T
16	CAGCGTGTCCGGCGAGGGCG	T	81	GATGCCGTTCTTCTGCTTGT	NT
17	AGCGTGTCCGGCGAGGGCGA	T	82	CAAGATCCGCCACAACATCG	T
18	CGCCGGACACGCTGAACTTG	NT	83	ATCCGCCACAACATCGAGGA	T
19	GGCGAGGGCGATGCCACCTA	T	84	TGCCGTCCTCGATGTTGTGG	NT
20	GGCATCGCCCTCGCCCTCGC	NT	85	CGCTGCCGTCCTCGATGTTG	NT
21	CTGAAGTTCATCTGCACCAC	T	86	TACCAGCAGAACACCCCCAT	T
22	CAGGGTCAGCTTGCCGTAGG	NT	87	CAGAACACCCCCATCGGCGA	T
23	CTTCAGGGTCAGCTTGCCGT	NT	88	GGTGTTCTGCTGGTAGTGGT	NT
24	CCGGCAAGCTGCCCGTGCCC	T	89	TGGGGGTGTTCTGCTGGTAG	NT
25	GGTGGTGCAGATGAACTTCA	NT	90	CGCCGATGGGGGTGTTCTGC	NT
26	CGGTGGTGCAGATGAACTTC	NT	91	CACGGGGCCGTCGCCGATGG	NT
27	GGGCACGGGCAGCTTGCCGG	NT	92	GCACGGGGCCGTCGCCGATG	NT
28	CCAGGGCACGGGCAGCTTGC	NT	93	AGCACGGGGCCGTCGCCGAT	NT
29	CTCGTGACCACCCTGACCTA	T	94	CAGCACGGGGCCGTCGCCGA	NT
30	ACGAGGGTGGGCCAGGGCAC	NT	95	GGTTGTCGGGCAGCAGCACG	NT
31	CACGAGGGTGGGCCAGGGCA	NT	96	TGGTTGTCGGGCAGCAGCAC	NT
32	GTGGTCACGAGGGTGGGCCA	NT	97	GTGGTTGTCGGGCAGCAGCA	NT
33	GGTGGTCACGAGGGTGGGCC	NT	98	GTGCTCAGGTAGTGGTTGTC	NT
34	GTCAGGGTGGTCACGAGGGT	NT	99	GGTGCTCAGGTAGTGGTTGT	NT
35	GGTCAGGGTGGTCACGAGGG	NT	100	CGGACTGGGTGCTCAGGTAG	NT
36	GTAGGTCAGGGTGGTCACGA	NT	101	TCAGGGCGGACTGGGTGCTC	NT
37	CGTAGGTCAGGGTGGTCACG	NT	102	GTCTTTGCTCAGGGCGGACT	NT
38	CTGCACGCCGTAGGTCAGGG	NT	103	GGTCTTTGCTCAGGGCGGAC	NT
39	GCACTGCACGCCGTAGGTCA	NT	104	CAACGAGAAGCGCGATCACA	T
40	AGCACTGCACGCCGTAGGTC	NT	105	GTTGGGGTCTTTGCTCAGGG	NT
41	GCTGAAGCACTGCACGCCGT	NT	106	CTCGTTGGGGTCTTTGCTCA	NT
42	GCTTCATGTGGTCGGGGTAG	NT	107	TCTCGTTGGGGTCTTTGCTC	NT
43	CGTGCTGCTTCATGTGGTCG	NT	108	GCGCGATCACATGGTCCTGC	T
44	TCGTGCTGCTTCATGTGGTC	NT	109	TGTGATCGCGCTTCTCGTTG	NT
45	GTCGTGCTGCTTCATGTGGT	NT	110	ATGTGATCGCGCTTCTCGTT	NT
46	TTCAAGTCCGCCATGCCCGA	T	111	CATGTGATCGCGCTTCTCGT	NT
47	AGAAGTCGTGCTGCTTCATG	NT	112	CTGGAGTTCGTGACCGCCGC	T
48	CATGCCCGAAGGCTACGTCC	T	113	TGGAGTTCGTGACCGCCGCC	T
49	GACGTAGCCTTCGGGCATGG	NT	114	ACCGCCGCCGGGATCACTCT	T
50	CTGGACGTAGCCTTCGGGCA	NT	115	CGCCGGGATCACTCTCGGCA	T
51	GGAGCGCACCATCTTCTTCA	T	116	CGGCGGTCACGAACTCCAGC	NT
52	CGCTCCTGGACGTAGCCTTC	NT	117	GCCGAGAGTGATCCCGGCGG	NT
53	GCGCTCCTGGACGTAGCCTT	NT	118	CATGCCGAGAGTGATCCCGG	NT
54	ACCATCTTCTTCAAGGACGA	T	119	GTCCATGCCGAGAGTGATCC	NT
55	TGAAGAAGATGGTGCGCTCC	NT	120	CGGTAGGCGTGTACGGTGGG	T
56	CAACTACAAGACCCGCGCCG	T	121	AAATGGGCGGTAGGCGTGTA	T
57	GCCGTCGTCCTTGAAGAAGA	NT	122	CATTGACGCAAATGGGCGGT	T
58	CCGCGCCGAGGTGAAGTTCG	T	123	CTCCGCCCCATTGACGCAAA	T
59	CGCGCCGAGGTGAAGTTCGA	T	124	GTTTTGGCACCAAAATCAAC	T
60	GAAGTTCGAGGGCGACACCC	T	125	CTACCGCCCATTTGCGTCAA	NT
61	CTCGAACTTCACCTCGGCGC	NT	126	ACCGCCCATTTGCGTCAATG	NT
62	CCTCGAACTTCACCTCGGCG	NT	127	GCCCATTTGCGTCAATGGGG	NT
63	GTCGCCCTCGAACTTCACCT	NT	128	CAAACTCCCATTGACGTCAA	NT
64	GGTGAACCGCATCGAGCTGA	T	129	TCCCATTGACGTCAATGGGG	NT
65	GTGAACCGCATCGAGCTGAA	T			

gRNA: guide RNA, No.: number, NT: Non-template, T: Template.

## Data Availability

The data of this study can be provided to researchers from the corresponding author upon reasonable request.

## Abbreviations

ceRNAs	Competing endogenous RNAs
CRISPRi	CRISPR interference
dsDNA	Double strand DNA
Dox	Doxorubicin
GN	Glomerulonephritis
HC	Healthy control
IC	Immune complexes
i.p.	intraperitoneally
LV	Lentiviral vector
lncRNA	Long noncoding RNAs
LN	Lupus nephritis
miRNA	microRNAs
MNCs	mononuclear cells
PBS	Phosphate-buffered saline
qRT-PCR	quantitative real-time polymerase chain reaction
RT	Reverse transcription
SEM	standard error of the mean
SLE	Systemic lupus erythematosus
SLEDAI	SLE disease activity index
TCR	T cell receptor
TUNEL	Terminal deoxynucleotidyl transferase dUTP nick end labeling
UPC	Urine protein concentration
